# Using the force: STEM knowledge and experience construct shared neural representations of engineering concepts

**DOI:** 10.1038/s41539-020-0065-x

**Published:** 2020-05-18

**Authors:** Joshua S. Cetron, Andrew C. Connolly, Solomon G. Diamond, Vicki V. May, James V. Haxby, David J. M. Kraemer

**Affiliations:** 1 Department of Psychology, Harvard University, Cambridge, MA 02138 USA; 2 Department of Education, Dartmouth College, Hanover, NH 03755 USA; 3 Department of Psychological and Brain Sciences, Dartmouth College, Hanover, NH 03755 USA; 4 Thayer School of Engineering, Dartmouth College, Hanover, NH 03755 USA

**Keywords:** Human behaviour, Learning and memory

## Abstract

How does STEM knowledge learned in school change students’ brains? Using fMRI, we presented photographs of real-world structures to engineering students with classroom-based knowledge and hands-on lab experience, examining how their brain activity differentiated them from their “novice” peers not pursuing engineering degrees. A data-driven MVPA and machine-learning approach revealed that neural response patterns of engineering students were convergent with each other and distinct from novices’ when considering physical forces acting on the structures. Furthermore, informational network analysis demonstrated that the distinct neural response patterns of engineering students reflected relevant concept knowledge: learned categories of mechanical structures. Information about mechanical categories was predominantly represented in bilateral anterior ventral occipitotemporal regions. Importantly, mechanical categories were not explicitly referenced in the experiment, nor does visual similarity between stimuli account for mechanical category distinctions. The results demonstrate how learning abstract STEM concepts in the classroom influences neural representations of objects in the world.

## Introduction

Learning changes perception. Over the course of learning, advanced students and experts grow to see the world differently from novice learners as a function of their knowledge and prior experience (e.g., chess^1^, physics^2^). For example, seminal work in cognitive psychology has demonstrated that when given the same set of physics practice problems, individuals with advanced knowledge of physics categorize the problems according to the abstract concept knowledge underlying each problem, whereas novices categorize problems according to the perceivable surface-level features of each problem^2^. Although a large body of neuroscience research has focused on the neural representation of visually perceivable categories^3–6^, the way in which abstract STEM concept knowledge is represented in the brain remains less explored. Here, using a cross-sectional design, we investigate how prior classroom-based and lab-based learning experiences influence the neural patterns that represent abstract conceptual categories when presented with naturalistic stimuli.

To date, only a few neuroimaging studies have investigated physics and engineering concept knowledge^7–10^. Results of this research implicate dorsal stream regions—including motor cortex—in the explicit retrieval of task-specific physics knowledge. However, in a naturalistic test of conceptual understanding, an important outcome of successful learning should be the ability to observe real-world stimuli and implicitly retrieve conceptual category information not available to novices^1,2^. Because prior fMRI studies of physics knowledge have focused on explicit retrieval of information about non-naturalistic stimuli, the question of how abstract physics and engineering knowledge is implicitly activated by real-world stimuli remains unresolved.

Within other concept domains, numerous fMRI studies have tied both explicit and implicit visual categorization—including categories based on real-world stimuli—to patterns of brain activity in the ventral visual stream^3–6,11^. For example, Connolly et al.^3^ have demonstrated that patterns of neural activity in ventral occipitotemporal cortex can reliably classify images of animals (viewed during an incidental task) into abstract conceptual categories. In the present study, we apply this analytical approach to the domain of mechanical engineering. Our aim is to identify patterns of brain activity associated with real-world stimuli that implicitly reflect learned abstract mechanical category information.

In pursuit of this aim, we recruited two groups of participants: a group of undergraduate mechanical engineering students with knowledge drawn from engineering lecture courses and hands-on laboratory experiences, and a control group of their peers: novice students at the same university matched for educational attainment. (Note that engineering students at the present institution do not have separate admissions criteria from students in other disciplines; the distinction between novice and engineering students in the present study is simply that engineering students had completed certain engineering and physics courses, whereas novice students had not elected any advanced courses in engineering or physics.) Both groups performed an fMRI concept knowledge task in which participants evaluated the Newtonian forces acting on a set of real-world structures. Using multivariate pattern analysis (MVPA) of neuroimaging data, we first identified convergent patterns of neural activity among engineering students and among novices. Then, for each group, we performed an informational network analysis^7^, a variant of representational similarity analysis (RSA^12^), to query those activity patterns for the presence of concept knowledge information about the mechanical categories of structures. As a control, we also evaluated the contribution of bottom-up perceptual information to these neural patterns using a forward-encoding model of primary visual cortex (HMAX^13^). In this way, we identified reliable neural patterns reflective of mechanical category knowledge elicited by real-world stimuli. By comparing these patterns across the two groups of students, our results reveal the influence of prior learning experiences on the neural representation of abstract STEM concepts.

## Results

### Engineering students demonstrate knowledge of Newtonian forces

**Fig. 1:**
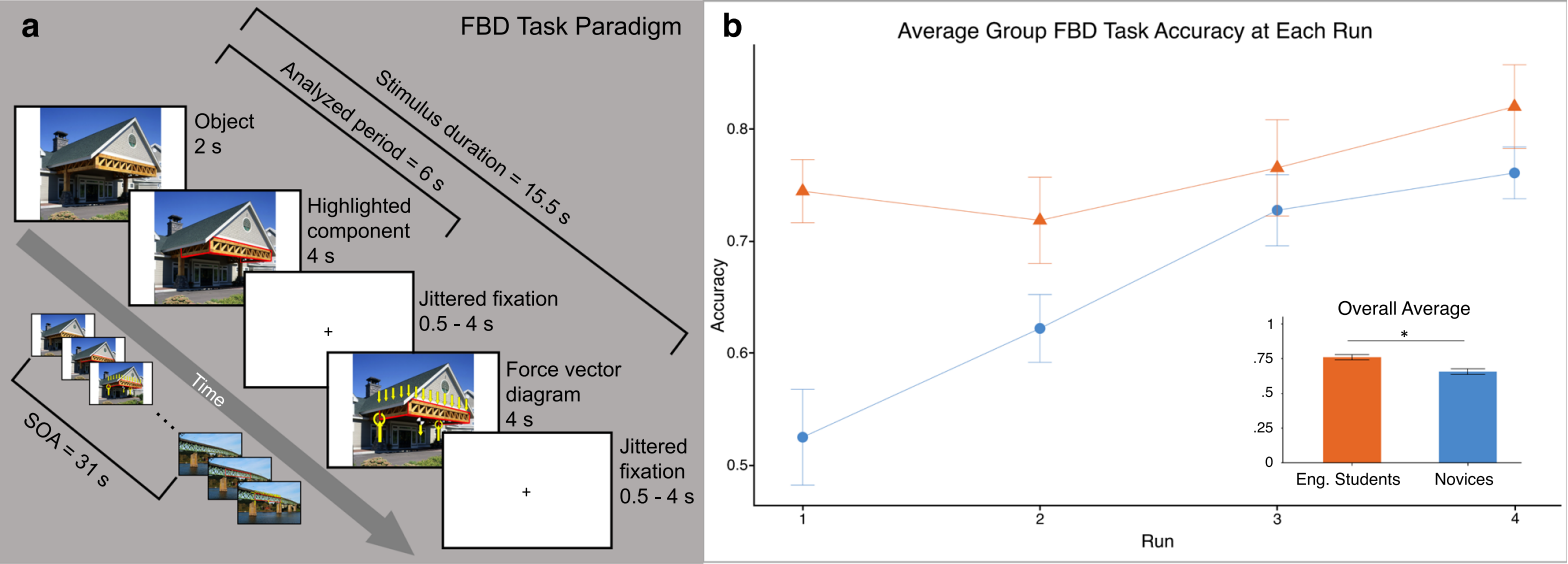
Engineering students significantly outperformed novices on the FBD task at the first fMRI run as well as on average. **a** FBD task structure for the fMRI paradigm. **b** FBD task results by group. By the final fMRI run, novices improved enough on the FBD task that they no longer significantly differed from engineering students in performance. Error bars indicate standard error of the mean. Note: The photos used in this figure are credited to Vermont Timber Works Inc. (the building awning stimulus example) and Steve Morgan (the truss bridge example stimulus). These images were edited and reused here with permission under the Creative Commons Attribution-ShareAlike 3.0 Unported license (https://creativecommons.org/licenses/by-sa/3.0/legalcode). Edits to these images were made by the authors, including the red highlighting and force arrow labels that were applied for use in the free body diagram task. All other elements of Fig. 1 were created by the authors.

Figure 1 shows the trial structure and behavioral results from the free body diagram (FBD) task that participants completed during fMRI scan. The FBD task required participants to evaluate the Newtonian forces interacting with real-world structures (see “Methods” section for a complete description of the FBD task). On average, engineering students significantly outperformed novices on this task (*M*_eng_ = 76%, *M*_nov_ = 66%, *t*(29) = 2.44, *p* = 0.02; Fig. 1b inset). A linear mixed-effects model revealed significant main effects of both run (*β* = 0.03, SE = 0.01, *p* = 0.01) and group (engineering students > novices, *β* = 0.24, SE = 0.06, *p* = 0.0005). There was also a significant two-way interaction (*β* = 0.05, SE = 0.01, *p* = 0.0005) in which novices improved more than engineering students over time (∆_eng_ = 7.55%, ∆_nov_ = 23.61%, *t*(29) = 3.47, *p* = 0.002). On the individual runs, the greatest difference in performance between groups was at run 1 (*M*_eng_ = 74%, *M*_nov_ = 53%, *t*(29) = 4.18, *p* = 0.0002). By run 4, both groups demonstrated mastery of the FBD task, and did not perform significantly differently from each other (*t*(29) = 1.34, *p* = 0.19).

### Overview of neural results

**Fig. 2:**
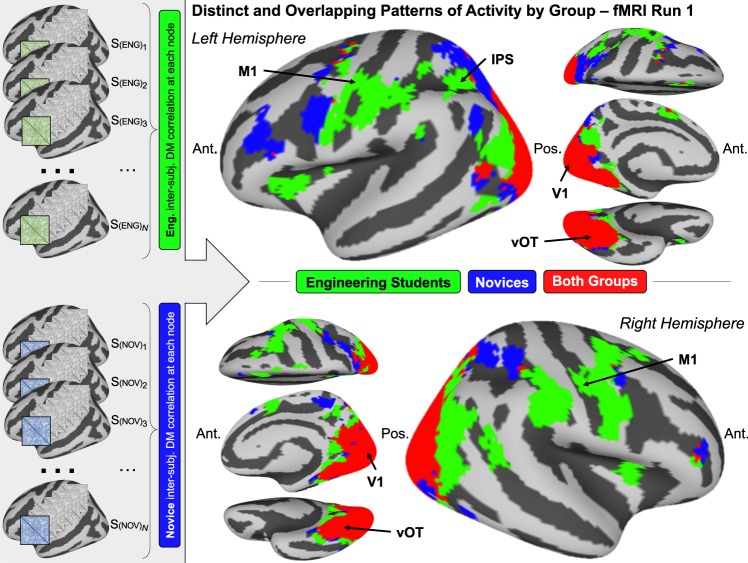
Novices and engineering students show distinct and overlapping patterns of neural activity. The schematic to the left describes the analytical procedure: First, in each subject’s brain, we computed a data-driven neural dissimilarity matrix (DM) at each surface node, consisting of the pairwise comparisons between stimulus items. Then, these individual subjects’ node-level DMs were correlated across subjects within each group. A noise threshold was applied using the negative extent of the intersubject correlations as a chance estimate. Finally, results were subjected to a spatial cluster correction on the cortical surface, retaining only clusters of at least five contiguous surface nodes. The brain surface maps on the right show regions where intersubject DM correlations were above the chance threshold for engineering students only (green), novices only (blue), and both groups (red). Anatomical reference labels have been added for M1, vOT, intraparietal sulcus (IPS), and calcarine sulcus (V1). Engineering students showed unique representational convergence in motor regions including M1, as well as ventral PFC and inferior parietal regions. Novice-specific regions included more anterior frontal regions and more dorsal parietal regions. Groups overlapped in representational convergence in occipital regions, including vOT until the most anterior regions.

**Fig. 3:**
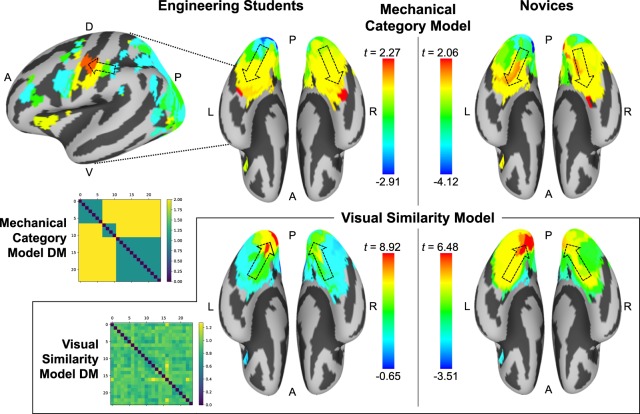
Neural patterns reflect mechanical category information within group-specific regions, whereas visual similarity information is represented in overlapping regions. Mechanical category informational gradients for engineering students (top left) and novices (top right), accompanied by the model DM for the mechanical categories of the stimuli. Engineering students showed a gradient of increasing mechanical category information moving from posterior-to-anterior in vOT, peaking in a bilateral anterior vOT informational network. Engineering students also showed a gradient of increasing mechanical category information in the dorsal stream moving toward M1. Novices showed no significant mechanical category information, but still showed a similar posterior-to-anterior gradient of increasing mechanical category information in the ventral stream. In the bottom panel, complementary results from the visual similarity analysis are shown, alongside the visual similarity model DM produced by HMAX. Both groups show peak visual similarity information in V1, with visual similarity information increasing along an anterior-to-posterior gradient in the ventral stream (the opposite direction from the mechanical category information gradient).

The neural results discussed here will consider only fMRI results from the first fMRI run. Task performance at fMRI run 1 maximally reflects the variance in prior knowledge and experience among participants. Consequently, fMRI data from run 1 are the most likely to show distinct patterns of brain activity associated with concept knowledge for engineering students compared to novices, if such a distinction should occur at all. In contrast, by fMRI run 4 we observe that participants from both groups have mastered the FBD task. It is therefore difficult to dissociate the effects of task-specific learning from the effects of prior knowledge and experience in any analysis of fMRI data from run 4. Thus, neural data from run 1 are analyzed here to identify the distinct and overlapping patterns of neural activity by group (Fig. 2) and the neural patterns reflecting mechanical and visual information by group (Fig. 3).

### Novices and engineering students show distinct and overlapping patterns of neural activity

In order to identify whether engineering students and novices showed unique patterns of brain activity when considering the stimulus items during the FBD task, we used a data-driven multivariate intersubject correlation analysis to discover any brain regions where participants within a group showed convergent neural representations of the stimulus items. For each individual participant at every surface node in the brain, we computed a dissimilarity matrix (DM) from the pairwise comparisons of item-level beta estimates at each node. Then, separately for each group, we computed the average (*z*-transformed) intersubject correlations of the DMs at each node. Finally, we used the negative extent of the correlation results as an estimate of the range of correlation values attributable to chance, resulting in a noise threshold of *z* = 0.02 (see Supplementary Methods and Supplementary Fig. 1 for a validation of the noise threshold using permuted null distributions of intersubject correlation values). For each group, nodes with an average intersubject DM correlation of *z* > 0.02 (cluster-corrected) are shown in Fig. 2, alongside a schematic of the analytical pipeline described here (whole-brain maps of the average intersubject correlation values before thresholding are shown in Supplementary Fig. 2). Importantly, this analysis was entirely data-driven; at no point was an a priori representational model introduced. (This analysis is described in full detail in the “Methods” section; see the subsection “Multivariate neural pattern analysis”, steps 1 and 2).

Out of 20,484 total nodes across the brain surface, there were 1,863 nodes where only engineering students showed above-threshold intersubject correlations (Fig. 2, green), 842 nodes where only novices showed above-threshold intersubject correlations (Fig. 2, blue), and 2,426 nodes where both groups show above-threshold intersubject correlations (Fig. 2, red). Representational convergence among engineering students was notably localized to motor regions including primary motor cortex (M1), portions of ventral prefrontal cortex (PFC), and inferior parietal regions. Representational convergence among novices was predominantly localized to more anterior frontal regions and superior parietal regions. The representational convergence maps for the two groups overlapped in regions including visual cortex, dorsal occipital cortex, and ventral occipitotemporal (vOT) cortex, although the groups remained distinct in the most anterior representations in vOT.

### Neural patterns reflect mechanical category knowledge in engineering students

To unpack the neural distributions of mechanical and visual information for each group within the regions of distinct and overlapping neural representations identified in Fig. 2, we computed an informational network analysis (see subsection “Multivariate neural pattern analysis”, steps 3 and 4 in the “Methods” section). First, for each group, we identified networks of brain regions within each of the distinct and overlapping areas with similar representations for the stimulus items, independent of any a priori representational model. Within the regions of high representational convergence among engineering students, a split-half repeated cross-validation procedure yielded 65 informational networks for engineering students and 67 informational networks for novices. Within the regions of high representational convergence for novices, the same procedure yielded 46 informational networks for engineering students and 28 informational networks for novices. Finally, within the regions where both groups showed high representational convergence, we identified 100 informational networks for each group.

After identifying the informational networks at the group level, we returned to the level of the individual participant. We computed the average representational DM for each informational network separately for each participant. The informational network DMs for each participant were then correlated with a mechanical category information model DM, as well as a visual similarity model DM as a control analysis. The mechanical category DM was generated using the pairwise similarity ratings of a mechanical engineering expert, and delineated three discrete categories of mechanical structures into which the stimulus set could be divided: cantilevers, trusses, and vertical loads. The visual similarity model DM for the stimulus items was generated using the HMAX forward-encoding model of primary visual cortex. Finally, we computed a summary statistic for the mechanical and visual correlation results at the group level using one-sample *t* tests against zero.

**Fig. 4:**
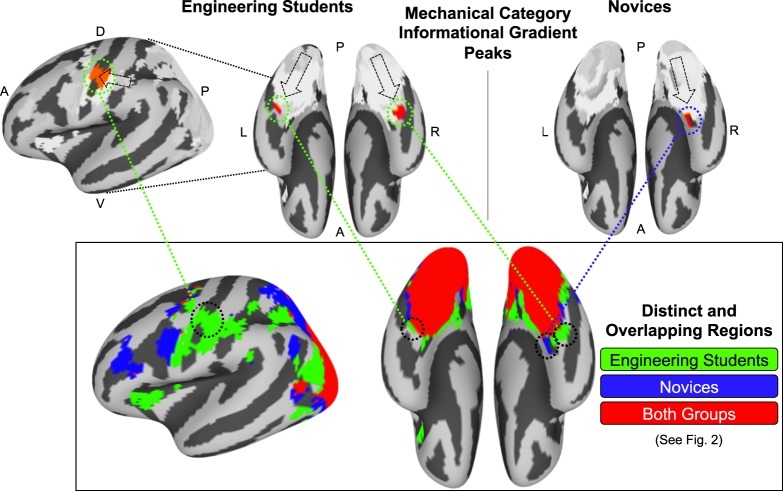
The peak regions for each mechanical category informational gradient shown in Fig. 3 each came from informational networks localized to group-specific regions (as identified in Fig. 2). Engineering students showed mechanical informational gradient peaks in a bilateral vOT informational network and an M1 informational network, each of which were localized to regions where engineers showed uniquely high representational convergence. Novices showed a mechanical informational gradient peak in a right vOT informational network, which was localized to a region where novices showed uniquely high representational convergence.

Figure 3 shows the cortical surface maps for mechanical category representations and the visual similarity representations for each group. Engineering students as a group showed a significant peak correlation with the mechanical category representation model in a bilateral anterior vOT informational network (*t*(15) = 2.27, *p* = 0.038), with a nonsignificant secondary peak in an M1 informational network (*t*(15) = 1.93, *p* = 0.073). Novices as a group showed a nonsignificant mechanical category representation peak in a right anterior vOT informational network (*t*(14) = 2.06, *p* = 0.058). Peak mechanical category representations for both groups came from group-specific distinct regions: the peak bilateral vOT and left M1 informational networks for engineering students came from areas with high representational convergence only among engineering students, suggesting that convergence among engineering students’ representations in those brain regions reflects a convergence upon mechanical representations. Figure 4 shows the peak informational networks for mechanical category information and visual information along the informational gradients shown in Fig. 3, identifying the distinct and overlapping regions from Fig. 2 to which those peak regions belonged. The peak right vOT informational network for novices came from an area with high representational convergence only among novices. Additionally, engineering students showed a gradient of increasing mechanical category information moving from posterior-to-anterior in the ventral stream in the direction of the vOT mechanical representational peak, as well as a similar gradient in the dorsal stream in the direction of M1. Novices showed a similar posterior-to-anterior gradient of increasing mechanical category representation along the ventral stream toward the nonsignificant mechanical representational peak in right vOT.

In an almost exact reversal from the results of the mechanical category analysis, the gradient of visual representations along the ventral stream for both groups showed visual similarity information increased from anterior-to-posterior along the ventral stream. Significant peak informational networks from the visual similarity analysis in both engineering students (*t*(15) = 8.92, *p* < 0.001) and novices (*t*(14) = 6.48, *p* < 0.001) occurred in primary visual cortex (V1), and were localized to regions where both groups exhibited high representational convergence. Thus, the visual similarity analysis affirms that the mechanical category representations cannot be accounted for by a model of item-level visual similarity alone.

**Fig. 5:**
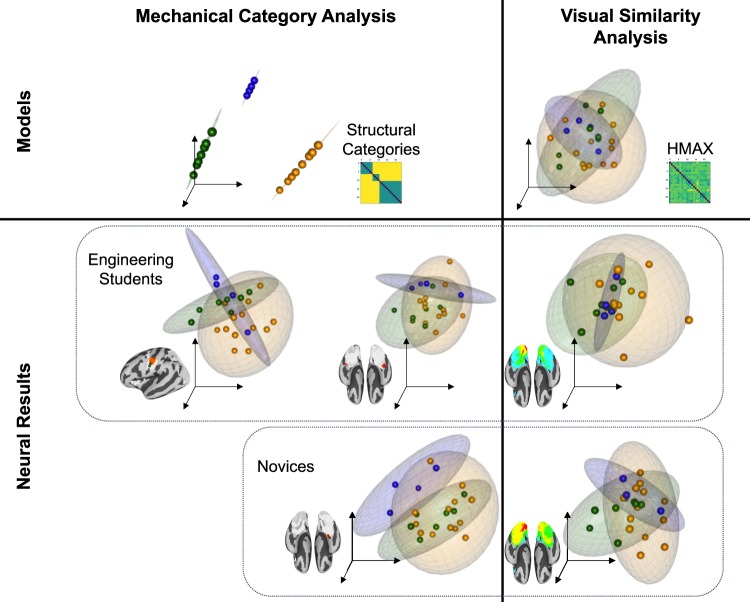
Descriptive visualization showing that the mechanical categories are most discernable among the peak informational networks from the mechanical category analysis. In contrast, the visual similarity model and the peak informational networks from the visual similarity analysis are not optimized to dissociate the mechanical categories. Item-level representations are shown for mechanical and visual representational models (top row), peak mechanical category informational networks for each group (bottom left), and peak visual similarity informational networks for each group (bottom right). Plots were generated using a three-dimensional embedding from a nonmetric multidimensional scaling algorithm. Points are color-coded by stimulus mechanical category: cantilevers (green), trusses (blue), and vertical loads (orange). The stimuli in each category are also surrounded by an ellipsoid of concentration identifying the region in which points belonging to that category are likely to occur with 95% probability under the assumption that points are drawn from a trivariate normal distribution.

As a descriptive visualization to contextualize the data-driven informational networks, the average informational network DMs for each peak informational network are displayed at the stimulus item level in Fig. 5, produced through multidimensional scaling into a three-dimensional embedding space. Concentration probability ellipsoids are plotted for each mechanical category (R package “rgl”^14^). This visualization highlights the mechanical category structure that is discernable even in the dimensionality-reduced representations for the mechanical category informational gradient peaks. By contrast, mechanical category structure is not as clearly identifiable within the visual informational gradient peak representations (nor in the visual similarity model representation itself).

### Evidence for task-specific learning by final fMRI run

**Fig. 6:**
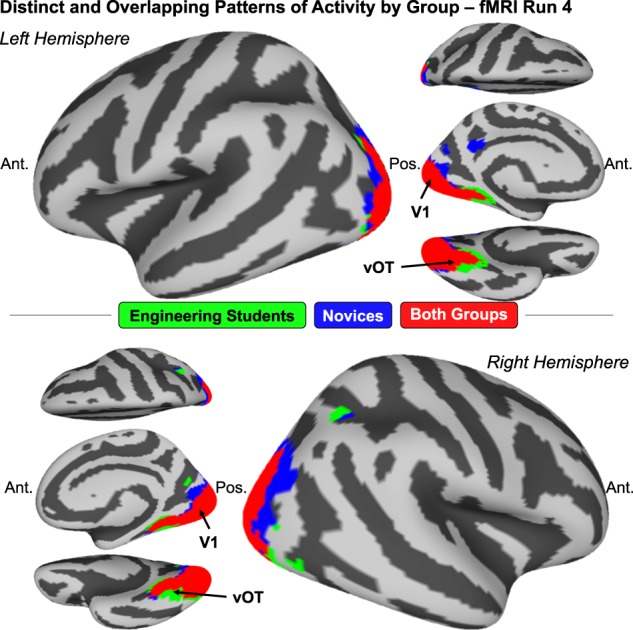
Participants in each group show less convergent neural activity at the last fMRI run. Brain surface maps of the results from fMRI run 4 for the same analysis applied to fMRI run 1 in Fig. 2. Compared to run 1, participants at run 4 showed fewer regions where intersubject DM correlations at run 4 were above the chance threshold for engineering students only (green), novices only (blue), and both groups (red). Each group had some representational convergence in right posterior parietal cortex, but otherwise convergent representations were found only in vOT. Engineering students showed more anterior representational convergence in vOT than novices. Groups continued to overlap in representational convergence in occipital regions.

As discussed above, the primary results of this study derive from the first fMRI run, because group differences in neural activity at the first fMRI run can be attributed to interindividual differences in prior knowledge of physics and engineering concepts. However, to provide a thorough treatment of the data, we also analyzed the results of the fourth and final fMRI run using the same multivariate intersubject correlation and RSA methods applied to fMRI run 1. The results of this analysis show that the neural and behavioral changes that take place between run 1 and run 4 of the fMRI task were likely predominantly associated with task-specific learning (i.e., identifying the now-familiar stimulus as either the correct or incorrect exemplar), and were not clearly associated with changes in conceptual knowledge over the course of the experiment. Participants did still show distinct neural representations between groups at run 4 (Fig. 6), although there were fewer overall convergent representations for each group (15 unique informational networks for engineering students, 2 unique informational networks for novices, and 43 informational networks for each group in regions of overlapping representational convergence). Furthermore, regions of representational convergence overall were almost entirely localized to vOT, with just one posterior parietal region of convergence for each group in the right hemisphere and no convergent representations in dorsal premotor or primary motor areas. Finally, results from the RSA for run 4 revealed that those convergent representations that did emerge for each group were no longer as strongly reflective of mechanical category information (but remained as strongly reflective of visual feature information as at run 1; Supplementary Fig. 3).

These results provide additional evidence that neural representations of Newtonian force concept knowledge are best identified at fMRI run 1. At the beginning of the experiment, the fact that there were group differences in neural representations at all *must* depend on the prior exposure to STEM education of the participants, because that is the only task-relevant dimension on which the participants varied upon beginning the experiment. By the end of the experiment, however, participants from both groups had improved on the behavioral task (although engineering students appear to encounter a ceiling effect; Fig. 1), despite not receiving any feedback about their task performance. This behavioral change is therefore not likely to derive from changes in conceptual understanding of physics and engineering, and indeed the observed changes in neural representational content are not consistent with an increase in the representation mechanical category information for either group (Fig. 6, Supplementary Fig. 3). Thus, the results from fMRI run 4 reveal that our multivariate analytical approach is particularly sensitive to the representations that participants are using at a given point during the fMRI experiment, and fMRI run 1 is the only point in the experiment where participants’ representations can be reasonably inferred to derive from differences in STEM concept knowledge.

## Discussion

The results of the present study demonstrate that classroom-based knowledge and experience inform the neural representations that students access when applying this knowledge in a naturalistic context. Our results show that engineering students exhibited convergent multivariate neural response patterns for real-world structures that strongly reflected information about the mechanical categories of those structures (Figs. 2–4). This finding is additionally striking because no explicit mention of any structural categories was made at any point during the experiment. Therefore, viewing of the task stimuli in the context of mechanical force evaluation was sufficient to activate the conceptual category information that engineering students had previously learned.

Results from the informational network analysis and the FBD task further support the interpretation that these neural patterns reflect abstract concept knowledge. At the beginning of the experiment, while engineering students explicitly demonstrated knowledge about mechanical force (Fig. 1b; high FBD task accuracy), their brain activity implicitly reflected mechanical category information about the structures considered in the task (Fig. 3). In contrast, novices showed poor explicit understanding of mechanical force during the first fMRI run (Fig. 1b; low FBD task accuracy), and their brain activity during this run reflected no significant mechanical category representations (Fig. 3). Taken together, these results demonstrate that (1) abstract concept knowledge that engineering students acquire in school is activated in response to real-world stimuli, and (2) this learned categorical knowledge can be identified within their multivariate neural response patterns.

The group differences in neural representation of engineering concept knowledge become more pronounced when compared with the results of the visual similarity control (HMAX) analysis. Unlike mechanical category information, visual feature information was significantly represented in brain regions where both engineering students and novices showed convergence in their multivariate neural response patterns (Figs. 2–4). These regions where both groups exhibited strong representations of item-level visual similarity were localized to posterior visual cortex, as expected. Notably, the anterior-to-posterior gradient of increasing visual similarity information in the ventral stream was opposite to the posterior-to-anterior gradient of increasing mechanical category information exhibited in the same region, leading us to conclude that visual and mechanical representations are supported by distinct brain regions, and that effective mechanical categorization of the stimulus items cannot be achieved using only the items’ visual feature similarities.

In terms of the localization of abstract mechanical force knowledge, it is interesting that we observe ventral stream results consistent with prior work^3–5,15^. Here, engineering students showed categorical concept knowledge represented in bilateral anterior vOT. We also observe a gradient of increasing mechanical category information in the dorsal stream, which is notable given prior research on the neural correlates of learned physics concepts (e.g., torque^8^, forces and causal motion^10^). In fact, these previous studies have localized explicit knowledge of linear and rotational force to many of the same specific regions we observe for the representation of mechanical force categories, including in particular M1, in which we observe representational convergence only for engineering students (Figs. 2 and 4). An intriguing possibility—to be explored by further research—is that the involvement of M1 in representing abstract concept knowledge for engineering students might be a function of their prior hands-on experiences in their lab-based coursework. Such an interpretation would be consistent with the studies cited above, as well as with our finding that novices show only premotor representations and not primary motor representations.

An additional aspect of the representational localization results that is worth highlighting is the comparison between the multivariate representational analyses employed here and traditional univariate contrasts of neural activity levels between the two participant groups. A supplementary univariate analysis (detailed in Supplementary Methods) revealed a convergent finding that engineering students’ cognition during these tasks is supported by motor and premotor and parietal regions in a way that is not observed for novices (Supplementary Fig. 4). However, that finding alone does not answer the question of where the item-level representations are housed and whether those representations differ between groups. Our multivariate analysis suggests that there is indeed a critical difference in item-level representations of conceptual information, but that those representational differences are most pronounced in the anterior ventral stream—a region that does not come out of the univariate general linear model (GLM). This difference between an analysis of global signal change and an analysis of representational geometry is precisely the advantage offered by MVPA techniques such as RSA.

One important feature distinguishing our current study from prior work is our use of naturalistic task stimuli in the form of real-world photographs, whereas these previous studies used fMRI tasks that were modeled after a specific lab task^8^ or that resembled taking a physics test^10^. Furthermore, the dorsal stream regions we observe have been consistently implicated in the extensive body of neuroimaging and neuropsychology investigations of the neural system underlying tool use and object knowledge^15–22^.

The research presented in the current study is not without certain limitations. As with any cross-sectional study, it is possible that some of the differences we observe derive from individual differences that are not related to participants’ educational background. However, there are several reasons why we believe extraneous individual differences are unlikely to have influenced the primary findings in this study. First, participants in this study were all enrolled in a university with among the most highly selective admissions criteria in the United States. As a result, factors such as general cognitive ability and even specific proficiencies (e.g., as represented by SAT math scores) are of a highly restricted range for both groups of participants. Moreover, unlike other institutions where technical or engineering schools have separate admissions criteria from the liberal arts college, the institution where the research was conducted requires identical admissions criteria for students entering into any discipline, including engineering. This reduces the likelihood of selection bias in terms of admission to the population from which participants were drawn.

In the present study, we demonstrated that STEM knowledge that students learned in school influenced their neural representations of real-world objects. The multivariate neuroimaging approach used here effectively differentiated groups of students on the basis of their prior knowledge and experience in the domain of mechanical engineering. Moreover, beyond merely demonstrating similar neural patterns among engineering students, our analyses revealed expert-like engineering knowledge within these patterns. Using this and other similar approaches, future research will elucidate the neural underpinnings of knowledge in other STEM concept domains, as well as investigate changes in neural representations of concept knowledge as STEM learning takes place over time.

## Methods

### Participants

Thirty-three students at Dartmouth College participated in this study. Two participants were dropped due to incomplete behavioral and scan data for a sample of *N* = 31 (*N*_female_ = 19; *M*_age_ = 20.65 years, SD = 1.70). Half of the participants (*N* = 15) had no background in engineering (referred to here as *novices*). Half (*N* = 16) were engineering students who, at the time of their participation, had taken or were nearly finished taking an engineering course intended for majors focusing on solid mechanics including a lab section (referred to here as *engineering students*). A lab-based course in advanced physics was a prerequisite for this engineering course. Of the engineering students, five had taken additional advanced structural engineering courses. Participants were recruited primarily through email listservs, provided informed consent prior to participation, and were compensated either with cash or curricular extra credit points. All protocols were approved by the Dartmouth Committee for the Protection of Human Subjects.

### Stimuli and design

Stimuli for the behavioral and scanner tasks (i.e., the similarity probe and the FBD task, detailed in the section “Procedure”) were 24 photographs of real-world, engineered structures. For each image, a component was selected to be the focal point of the FBD analysis, and that component was indicated with a red outline. For evaluation in the FBD task (described below), two more versions of each image were created, each labeled with arrows indicating the forces and moments acting on the component of interest. One arrow-labeled version was labeled correctly, and the other was labeled incorrectly, for use in the FBD task completed during scanning.

### Free body diagram (FBD) task

Figure 1a illustrates the concept knowledge task regarding the interaction of Newtonian forces with real-world structures. Our goal was to design a task that elicited knowledge of Newtonian force, but for which mechanical category information about the structures was incidental. Therefore, we never mentioned the mechanical category names during the task, nor did we even refer to the existence of categories in the task instructions or at any time during the experiment.

In the scanner, participants completed the following analytical task concerning the equilibrium state of a segment of interest in each of 24 images of real-world structures: Participants saw each structure image first for 2 s without any additional markings, and then for 4 s with the component of interest outlined in red. This was followed by a jittered fixation period during which participants had been previously instructed to imagine the forces and moments acting on the component of interest to maintain static equilibrium in the system. Functional imaging data collected from this fixation/consideration period were those used in all neural analyses. Finally, the component-highlighted image reappeared for 4 s, this time with arrows labeling the forces and moments either correctly or incorrectly. Participants had to assess whether the labeling was correct or incorrect based on the model they had imagined during the fixation period, and indicate their evaluation via button press during the 4-s window. This was followed by one more jittered fixation period, to round off the duration of the trial at exactly 15.5 s. Finally, each trial was separated by 15.5 s of fixation to establish a baseline for the fMRI analysis.

Each participant completed four runs of the FBD task, and each run included all 24 stimulus images. On each run, 12 images (50%) were correctly labeled. The specific images that were correctly or incorrectly labeled varied pseudo-randomly from run to run such that by the end of the experiment, every participant had seen both the correct and incorrect versions of every stimulus twice.

### Procedure

Participants completed two sessions within a period of at most 7 days: a behavioral session followed by functional magnetic resonance imaging (fMRI) scanner session. In the behavioral session, participants first completed a similarity ratings task where they rated the complete set of stimuli for nonspecific interitem similarity. They then completed two standardized tests of engineering and physics knowledge: the Statics Concept Inventory (SCI^23^) and the Force Concept Inventory (FCI^24^). Results of the similarity ratings task and the standardized evaluations are not discussed in the present study; see our prior work^7^ for analysis and discussion of these results.

In the fMRI session, all participants were given an introduction to the concepts of Newtonian force, static equilibrium, and FBDs. After viewing the concept primer twice, they were shown the 24-item experimental stimulus set for familiarization and completed practice trials of the FBD task with a separate set of practice stimuli. Participants then completed the FBD task in the scanner over the course of four functional runs. Feedback (“correct” or “incorrect”) was given for practice trials, but not for experimental trials. At the end of the scanner session, participants completed a post-scan version of the similarity ratings task (results not discussed here).

### Imaging acquisition parameters

Brain images were acquired using a 3T Philips Achieva Intera scanner with a 32-channel head coil, using gradient-echo echo-planar imaging. For functional scans, an 80 × 80 reconstruction matrix was used with a 240 mm^2^ FOV for whole-brain coverage over 42 transverse slices (Flip angle = 90°; TE = 35 ms; TR = 2500 ms; 3 mm^3^ voxels; no gap). Slices were acquired in an interleaved order. Data were collected over four functional runs, each run consisting of 298 volumes. Data from of these functional runs—the first and the last—were used in the present analyses. For each participant, a single high-resolution T1-weighted anatomical scan was also collected (TE = 3.72 ms; TR = 8.176 ms; voxel resolution = 0.938 × 0.938 × 1.0 mm).

### Functional image preprocessing

Preprocessing of fMRI data was carried out using the FSL FEAT software package^25,26^. First, for each participant, a high-resolution T1-weighted anatomical image was skull-stripped using the FSL brain-extraction tool^27^. Then, for each functional run, skull-stripping, motion correction, slice timing correction, prewhitening, and highpass temporal filtering (cutoff at 100 s) were applied to the EPI volumes. The functional images were then registered to the participant’s individual anatomical volume using the FSL linear registration tool^28–30^.

Next, in order to calculate beta-value estimates for each stimulus, an item-level univariate regression model was computed for each functional run using the GLM. To accomplish this, an explanatory variable was set up to model the brain activity associated with each individual stimulus. Activity was sampled from the initial 6 s of each trial, during which participants were shown an image of the stimulus and asked to imagine the Newtonian forces acting upon a given section of the structure (see Fig. 1a: *analyzed period*). This portion of the trial was separated from the remainder of the trial by a jittered fixation period, to allow for an unconfounded estimate of the BOLD signal. In order to model this brain activity separately from the rest of the trial, during which the participant responded via button press, a separate explanatory variable was set up to model brain activity associated with the response period (4 s per trial) combined across all trials. Brain activity associated with the response period was excluded from further analysis. The beta estimates associated with the individual stimuli (first 6 s of each trial) were then used in a multivariate analysis, described below.

In the final preprocessing step, we used the Freesurfer recon-all suite^31^; http://surfer.nmr.mgh.harvard.edu) to carry out cortical surface reconstruction for each subject-level T1-weighted image. These cortical surfaces were then transformed to Surface Mapping (SUMA) format^32^; http://afni.nimh.nih.gov/afni/suma/). Each participant’s cortical surface map was then fitted to standard mesh grids defined by an icosahedron with 32 linear divisions, yielding 20,484 nodes for the whole-brain cortical surface. Using sulcal alignment of each participant’s cortical surface to the common MNI template provided by Freesurfer, this inflated surface mapping allowed for anatomical correspondence between surface nodes across participants.

### Multivariate neural pattern analysis

Our goal was to determine whether the two groups of participants displayed unique neural activity patterns while processing the task stimuli, and to characterize and localize those patterns if they emerged. We employed a series of multivariate neuroimaging analyses detailed below, using the fMRI data collected during the first run of the FBD task (the point of maximal task performance disparity between the two groups). The analyses proceeded in four steps as follows:

Step 1: Identify emergent neural representations for each subject. First, we constructed for each subject an item-level dissimilarity matrix (DM) at every one of the 20,484 searchlight locations in the brain. This was achieved in python^33^ using the PyMVPA toolkit^34^ by calculating, for each searchlight node, every pairwise correlation of item-level beta estimates at that node. The result of this analytical step was a set of subject-level surface maps, where each surface node contained a DM reflecting that node’s representation of the stimulus set (Fig. 2, left panel).

Step 2: Intersubject correlations by group. To identify whether the two groups showed group-level convergence of neural representations, we then calculated intersubject correlations between the subject-level DM surface maps. The intersubject correlation analysis proceeded as follows:

1. We computed the pairwise intersubject DM correlation values within each group at every surface node on the brain.
2. We applied a Fisher *z*-transformation (using the hyperbolic arctangent) to each intersubject correlation value.
3. We computed the average intersubject correlation (now a *z* value) for each surface node. This yielded a whole-brain correlation map of average *z* values for each group at the first fMRI run (Supplementary Fig. 2).
4. In order to threshold the whole-brain maps for correlation values we could reasonably attribute to noise, we applied the following threshold: because a negative intersubject correlation value in this type of multivariate analysis is most likely due to noise, we used the negative extent of the observed intersubject correlation values as an estimate of the noise distribution of the data. For each whole-brain correlation map we computed, the negative extent of the data was approximately *z* = −0.02, so we applied a noise threshold of *z* > 0.02 to the correlation maps. This threshold was further validated using permuted null distributions^35^ (details of this permutation analysis are reported in Supplementary Methods).
5. Finally, to produce the visualization shown in Fig. 2, we compared the two group-level average intersubject correlation maps by simply overlaying them on a common cortical surface projection. Regions where both groups exhibited average intersubject correlation values of *z* > 0.02 were labeled as “overlapping” regions, while regions where only one group exhibited average intersubject correlation values of *z* > 0.02 were labeled as “distinct” regions. For visualization in Fig. 2, spatial clustering was also applied to the final cortical surface map using the AFNI SurfClust function^36^ to retain only clusters of at least five contiguous surface nodes.

The above steps were applied separately to DMs from the first and last fMRI runs.

Step 3: Informational network analysis. In order to query the brain for mechanical category information to identify any neural representations that reflected STEM concept knowledge, we performed an informational network analysis^7^. The analysis proceeded as follows: First, for each group, average node-level DMs were extracted from the distinct and overlapping brain regions defined in the intersubject correlation analysis in Step 2 (Fig. 2). Next, the average node-level DMs for each group were subjected to Ward hierarchical clustering separately for the engineering-student-specific, novice-specific, and overlapping regions shown in Fig. 2. The number of hierarchical clusters was determined using a split-half cross-validation procedure used by Connolly et al.^37^ testing cluster solutions from 2 to 100 clusters over 1000 repetitions^37^. The clusters identified for each group within each set of distinct and overlapping brain regions are designated as informational networks, because they are defined according to their shared informational content without reference to any external or a priori representational model.

Having defined the informational networks at the group level, an RSA was performed at the individual participant level on the representations within each informational network. Separately for each participant, an average DM was computed for every informational network. Next, the normalized correlations were calculated between each informational network DM and a model DM representing the mechanical structure category for each stimulus item. Mechanical category information for the stimulus set was determined in consultation with a field expert (author SGD), and was never explicitly identified or discussed during the experiment to any participant. Normalized correlations were computed by first normalizing each informational network DM as well as the mechanical category target DM, and then computing the dot product of each informational network DM and the mechanical category DM. Finally, within each group, participant-level correlation distributions (*z*-values) across the informational networks were subjected to a one-sample *t*-test against zero to identify the degree to which each informational network’s representation consistently correlated with the mechanical or visual DM across the members of a group.

The group-level results from the one-sample *t*-tests were projected onto the cortical surface, yielding a surface map for each group showing the gradient of mechanical category information represented across the informational networks for each group (Fig. 3). As a supporting visualization of the item-level representations in each informational network, the average informational network DMs for the peak regions of mechanical category information from each group were extracted, and item-level representations were rendered using 3-dimensional nonmetric multidimensional scaling (MDS; “smacof” R package^38^). Those item-level MDS representations are displayed in Fig. 5.

Step 4: Visual similarity control model. To validate our analysis of mechanical information from neural activity patterns, we performed a control analysis using a model of visual similarity between items in the stimulus set. For this control analysis, we computed a visual similarity model of the stimuli using a forward-encoding model of primary visual cortex (HMAX, C1 layer^13^). RSA was then applied over the same informational networks defined in Step 3, using the identical approach as for the mechanical category model but this time targeting the visual similarity model. The surface maps for each group showing the correlations between neural representations and the visual similarity model for each group are displayed in Fig. 3, and item-level representations in the peak visual similarity regions are displayed using multidimensional scaling alongside the mechanical category information peaks and the mechanical and visual model DMs in Fig. 5. (The mechanical and visual RSA analyses were additionally computed for the last fMRI run, and the results are displayed in Supplementary Fig. 3.)

## Supplementary information

Supplementary Information

nr-reporting-summary-cetron_kraemer_11_25_2019

## Data Availability

The data sets generated during the current study are available from the corresponding author on reasonable request.

## Code availability

The code generated during the current study is available from the corresponding author on reasonable request.
