# Supplementary material for: Using the force: STEM knowledge and experience construct shared neural representations of engineering concepts
Source: NPJ Sci Learn. 2020 May 18;5:6. doi: 10.1038/s41539-020-0065-x (PMC7235041; doi:10.1038/s41539-020-0065-x)
Supplement: Supplementary file 1 — Supplementary Information [file 41539_2020_65_MOESM1_ESM.pdf]

Supplement to Cetron et al.

"Using the Force: STEM Knowledge and Experience

Construct Shared Neural Representations of Engineering Concepts"

Supplementary Methods

Validating inter-subject correlation threshold with permuted null correlations. In order to further validate our inter-subject correlation noise threshold of $z > 0.02$, we computed a permuted null correlation distribution analysis. This analysis allowed us to generate permuted null distributions for each surface node of the brain based on each subject's node-level DMs, which we used to create a distribution of null inter-subject correlation maps. Analysis of these null distributions yielded noise boundary estimates of approximately $z = -0.013$ (lower bound) and $z = 0.014$ (upper bound). Based on these estimates, we conclude that our chosen noise threshold of $z > 0.02$ confidently excludes spurious null correlations from our inter-subject correlation analysis. The permutation analysis proceeded as follows (computed separately for fMRI runs 1 and 4):

1. For each subject individually, we randomly permuted the item labels within the DM at each surface node in the brain, yielding a whole-brain map of shuffled DMs for each subject.
2. We repeated steps 1-3 of the original analysis exactly as before (see Methods section in the main text), except now using the shuffled DMs for each participant rather than the true DMs. This yielded a whole-brain average *z*-value map of null inter-subject correlations for each participant group.
3. We repeated this process for 1000 sets of permuted subject-level DMs, generating 1000 whole-brain maps of null inter-subject correlations for each participant group.
4. To determine appropriate threshold values, we identified the *z*-values marking the percentiles for 2.5% and 97.5% of each surface node's 1000 null correlation values.

5. We took these percentile threshold values and plotted the distribution of upper-bound and lower-bound *z*-values across the whole brain for each group (Supplementary Figure 1). We take the extreme values (i.e., the maximum of the upper-bound distributions and the minimum of the lower-bound distributions) for each group to be our permuted null threshold values for the true inter-subject correlation maps.

Univariate general linear model (GLM) analysis. In addition to the multivariate analyses described in the main text, we performed a set of standard univariate contrast analyses using general linear models (GLM) with FSL FEAT (Jenkinson, Beckmann, Behrens, Woolrich, & Smith, 2012; Smith et al., 2004; Woolrich et al., 2009). First, individual participant-level contrasts were computed using a first-level GLM identifying changes in BOLD response from a fixation baseline to the stimulus image consideration period (Figure 1A, "Analyzed Period"). Next, group-level contrasts were computed using a second-level GLM comparing the images > baseline beta parameters between engineering students and novices. These analyses were computed separately for fMRI runs 1 and 4. Clusters of significant activity differences for each contrast were determined by FSL using a threshold of $z > 2.3$ with a corrected cluster significance threshold of $p = 0.05$ [1].

The only univariate GLM contrast to yield significant activity clusters was the contrast of engineering students > novices at fMRI run 1 (Supplementary Figure 4). Engineering students showed significantly greater activity than novices in the left intraparietal sulcus (IPS), dorsal premotor cortex (PMd), and occipital cortex. These clusters are consistent with prior literature suggesting engineering students engage left-lateralized dorsal regions including IPS and PMd during tasks involving physics concept knowledge [2].

Supplementary Figures

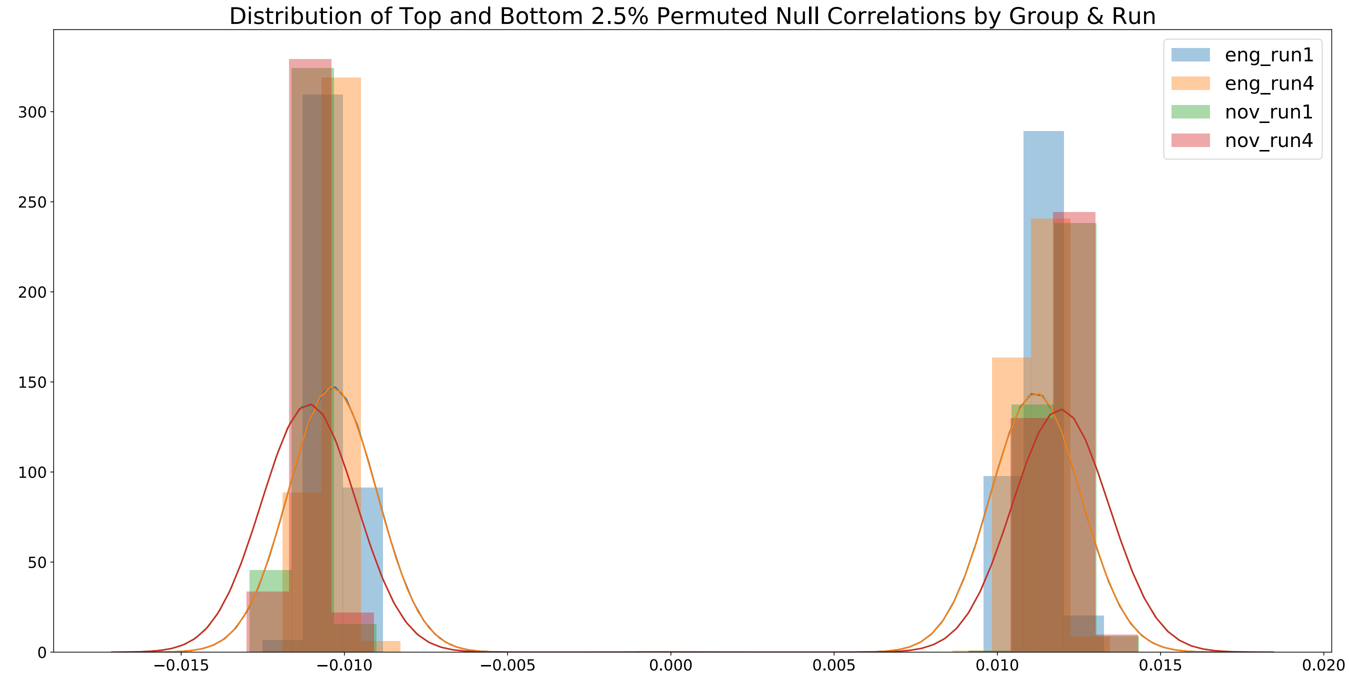

Supplementary Figure 1: Histograms of permuted null distributions of inter-subject correlations validate noise thresholding at $z > 0.02$. Extreme values from the top and bottom 2.5 percentiles of the permuted null distributions of inter-subject correlation values provide noise boundary estimates of $z = -0.013$ (lower bound) and $z = 0.014$ (upper bound). Lines represent interpolated curves over the histogram bins. (Data visualization created using Seaborn package in Python: https://seaborn.pydata.org [3])

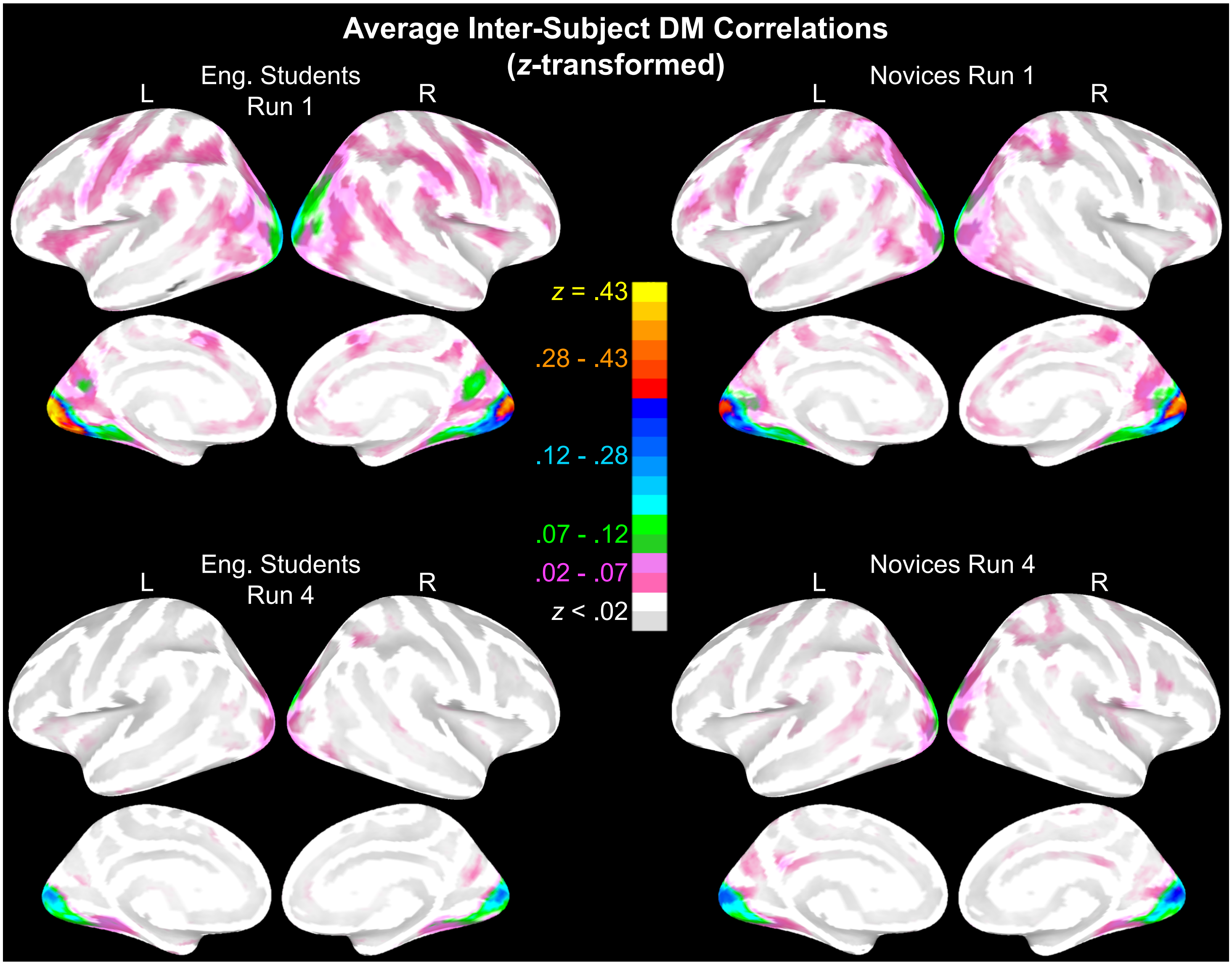

Supplementary Figure 2: <u>Whole-brain maps of average inter-subject correlation values by group and run.</u> Values below the $z > 0.02$ threshold are shown in grayscale; other correlation values are shown in color as indicated in the color scale. Above-threshold regions correspond to the red, blue, and green regions in Figure 2 (for run 1) and Figure 6 (for run 4) of the main text. Green regions in Figures 2 and 6 are above-threshold regions for engineering students and not for novices. Blue regions in Figures 2 and 6 are above-threshold regions for novices and not engineering students. Red regions in Figures 2 and 6 are above-threshold regions for both groups.

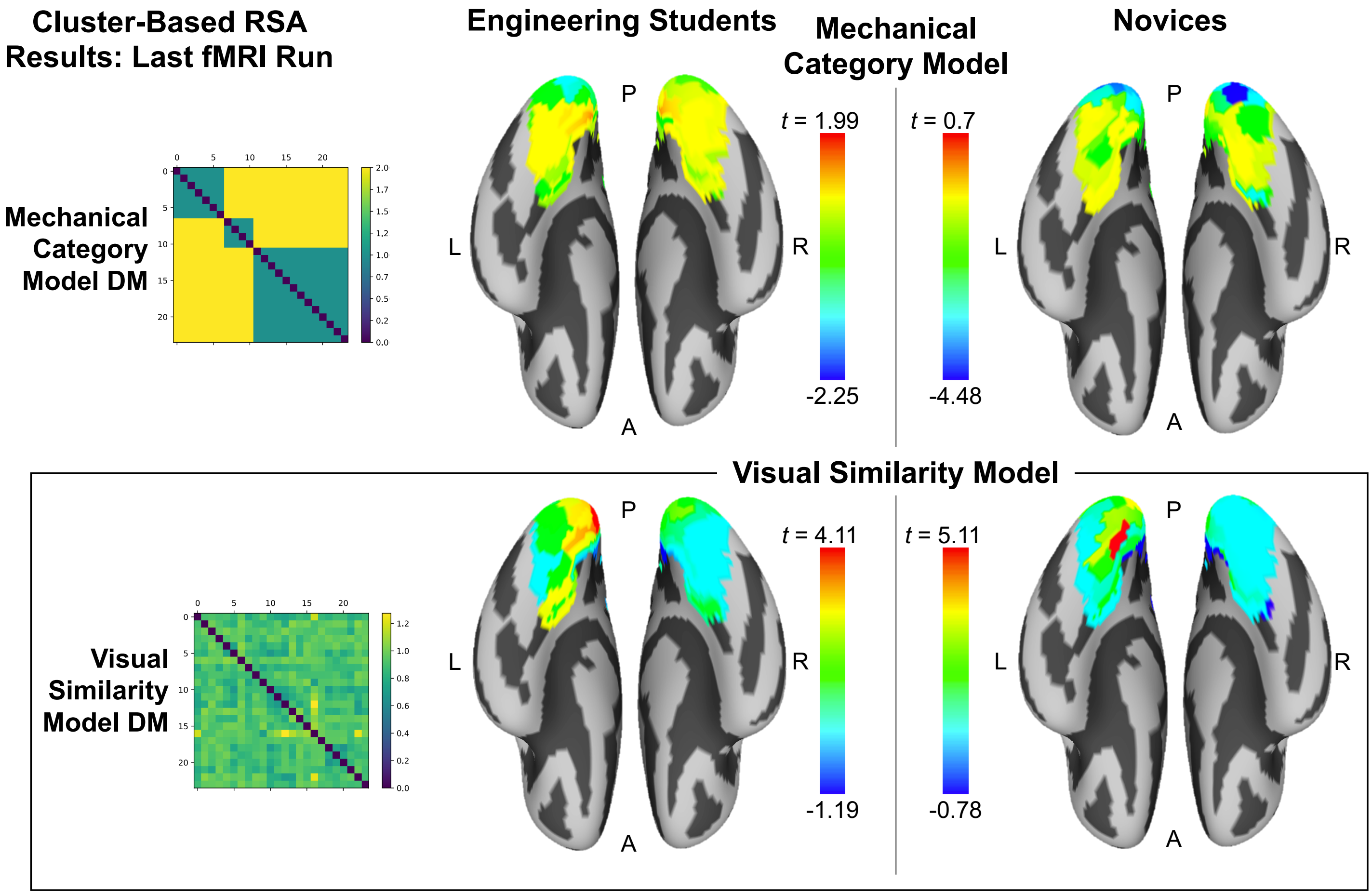

Supplementary Figure 3: Cluster-based RSA of fMRI run 4 shows persistent visual representations but diminished mechanical category representations. Results of the mechanical category RSA (top) and visual similarity RSA (bottom) performed on informational network DMs from fMRI run 4 show that visual feature information continues to be strongly represented by the HMAX model DM. The mechanical category model DM is more weakly represented by informational networks at run 4, and is especially weakly represented among novices. This result supports the interpretation that participants are not relying on conceptually-relevant mechanical category information at run 4, given the sensitivity of the multivariate approach to the specific representations participants are using at particular points in the experiment.

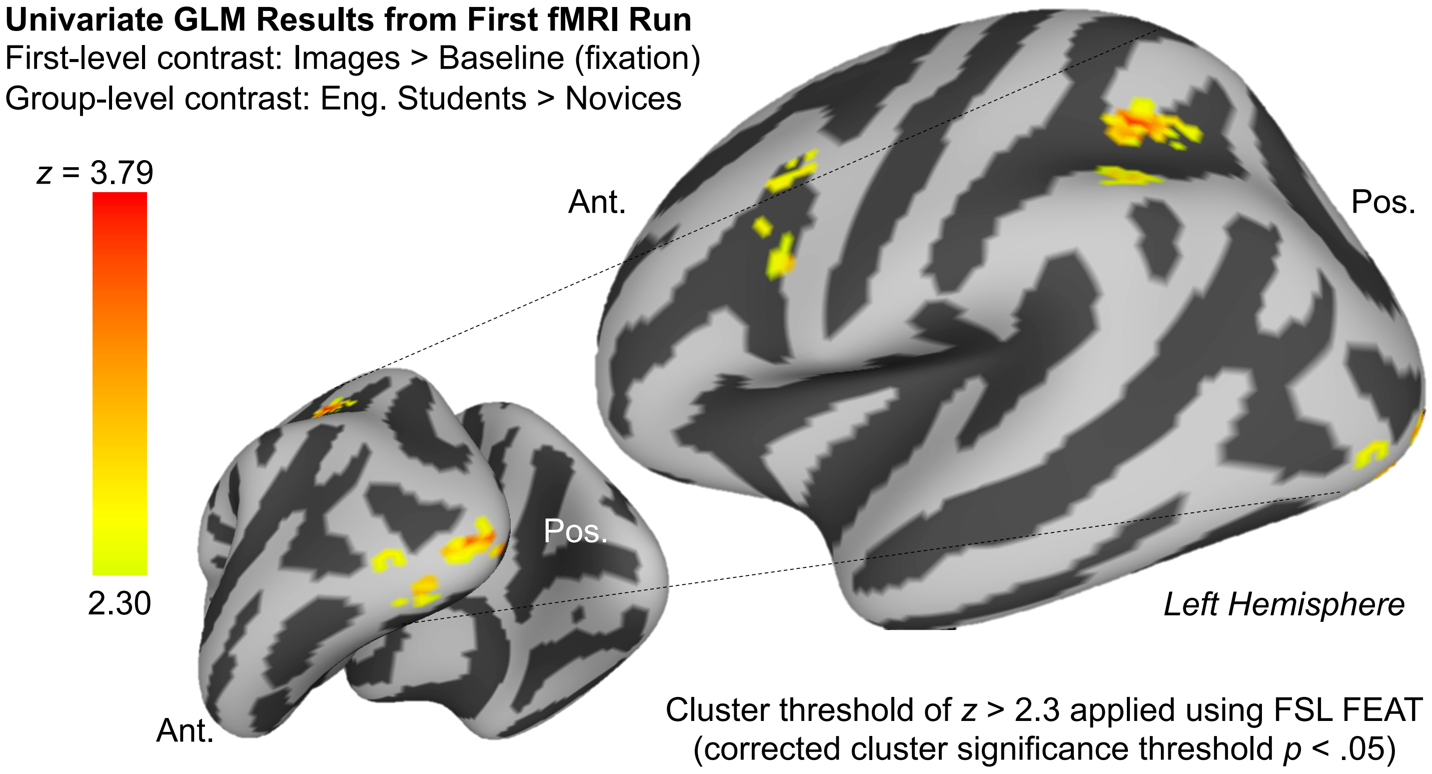

Supplementary Figure 4: Univariate contrasts reveal greater task-related neural activity in engineering students than novices. Engineering students showed greater activity in response to task stimuli than novices at fMRI run 1, specifically in clusters within the left intraparietal sulcus (IPS), dorsal premotor cortex (PMd), and occipital cortex. These clusters are consistent with prior literature suggesting engineering students engage left-lateralized dorsal regions including IPS and PMd during tasks involving physics concept knowledge.

Supplementary References

1. Worsley, K. Statistical Analysis of Activation Images. *Funct. MRI Introd. Methods* **14**, (2001).
2. Kontra, C., Lyons, D. J., Fischer, S. M. & Beilock, S. L. Physical experience enhances science learning. *Psychol. Sci.* **26**, 737–749 (2015).
3. Waskom, M. seaborn: statistical data visualization. https://seaborn.pydata.org/.
